# Vitamin K – a scoping review for Nordic Nutrition Recommendations 2023

**DOI:** 10.29219/fnr.v67.10260

**Published:** 2023-10-23

**Authors:** Arja T Lyytinen, Allan Linneberg

**Affiliations:** 1 Institute of Public Health and Clinical Nutrition, University of Eastern Finland, Kuopio, Finland; 2 Center for Clinical Research and Prevention, Bispebjerg and Frederiksberg Hospital, Copenhagen, Denmark

**Keywords:** vitamin K, phylloquinone, menaquinone, dietary recommendations

## Abstract

Vitamin K occurs in dietary supply in two major forms: phylloquinone (vitamin K1) and menaquinones (collectively referred as vitamin K2). Phylloquinone is derived from plants. There are at least 10 forms of menaquinones varying in chain length and they are produced by bacteria except menaquinone-4. Menaquinone-4 is formed from phylloquinone or other menaquinone forms. Phylloquinone is considered to be the major contributor and menaquinones are thought to contribute less to vitamin K intake in Western diets. However, less is known about the content of menaquinones than phylloquinones in foods.

Vitamin K is known to function as an enzymatic cofactor in the gamma-carboxylation of vitamin K dependent proteins (VKDPs). Hepatic VKDPs are involved in coagulation. Extrahepatic VKDPs have a role e.g. in bone health and vascular calcification. However, the amount of vitamin K needed for optimal functioning of the different VKDPs is not known.

## Popular scientific summary

- Vitamin K is a fat-soluble vitamin and includes two forms, phylloquinone (vitamin K1) and menaquinones (vitamin K2), of which phylloquinone is considered the main form in Western diets.
- Dietary sources of phylloquinone include leafy green vegetables, and certain vegetable oils.
- Vitamin K is essential for activation of vitamin K dependent proteins which are involved in blood clotting, bone metabolism and calcification.
- Vitamin K deficiency can cause bleeding and haemorrhage.
- The current evidence for preventive effects on chronic diseases is limited.

Vitamin K is the collective term for lipid-soluble compounds with vitamin K activity and having the common 2-methyl-1,4-naphtoquinone ring structure. Vitamin K occurs naturally in two forms: phylloquinone, or vitamin K1 (2-methyl-3- phytyl-1,4-naphtoquinone) and menaquinones, or collectively referred as vitamin K2 (multi-isoprenylquinones, several species). Phylloquinone is synthesised by plants, while the menaquinones are produced by bacteria except menaquinone-4. Menaquinone-4 is formed from phylloquinone or other menaquinone forms. Phylloquinone has been thought to be the major dietary form of vitamin K; however, the estimation of dietary intake of menaquinones is challenging as the food composition databases have limited information on menaquinone contents.

All forms of vitamin K function as an enzymatic cofactor in the gamma-carboxylation of vitamin K dependent proteins (VKDPs). There are several VKDPs of which the hepatic proteins needed in coagulation were first identified and later extrahepatic VKDPs involved in bone metabolism, calcification and other functions have been identified. The aim of this scoping review is to describe the totality of evidence for the role of vitamin K for health-related outcomes as a basis for setting and updating dietary reference values (DRVs) (Box 1).

Box 1 NNR2023 scoping reviews.

- This paper is one of many scoping reviews commissioned as part of the Nordic Nutrition Recommendations 2023 (NNR2023) project (1).
- The papers are included in the extended NNR2023 report but, for transparency, these scoping reviews are also published in Food & Nutrition Research.
- The scoping reviews have been peer reviewed by independent experts in the research field according to the standard procedures of the journal.
- The scoping reviews have also been subjected to public consultations (see report to be published by the NNR2023 project).
- The NNR2023 committee has served as the editorial board.
- While these papers are a main fundament, the NNR2023 committee has the sole responsibility for setting dietary reference values in the NNR2023 project.

## Methods

The review on vitamin K follows the protocol developed within the NNR2023 project (1). The sources of evidence used in the review follow the eligibility criteria described in the paper ‘The Nordic Nutrition Recommendations 2022 – Principles and methodologies’ (2).

The literature search contained the following terms (((‘vitamin k’[MeSH Terms] OR ‘vitamin k’[Title]) AND (((‘2011’[PDAT] : ‘3000’[PDAT]) AND Humans[Filter]) AND (review[Publication Type] OR systematic review[Publication Type] OR meta-analysis[Publication Type)))) AND ((Diet OR Dietary OR Food OR Nutrition OR Nutritional)). The search was conducted on 29.6.2021 using PubMed. The search yielded 237 articles, out of which 68 articles were selected to be read fully. Furthermore, European Food Safety Authority (EFSA) has published a review on vitamin K for setting up DRVs in 2017 (3). There were no *de novo* NNR2023 systematic reviews (SRs) or qualified SRs available for the topic.

## Physiology

Vitamin K is absorbed mainly in the small intestine through the pathway common to most dietary lipids (4). Absorption of phylloquinone is highly variable, with 80% of purified phylloquinone from supplements, but considerably less (3–50% of that absorbed from supplements) from plant foods (3). Absorption is dependent on food matrix and accompanying meal. Most importantly, absorption is enhanced by concomitant intake of dietary fat. Less is known about the absorption of dietary menaquinones than that of phylloquinone (5). Little is known about the absorption of menaquinones produced by gut bacteria in the large intestine. Absorbed vitamin K is transported by chylomicrons in the lymph and is primarily taken up in the liver (4). Vitamin K is bound to lipoproteins in the blood, the main carrier of phylloquinone being triglyceride rich lipoproteins. Phylloquinone is relatively short-lived in the blood compared to most menaquinones. Vitamin K is primarily stored in adipose tissue, but the total body pool of vitamin K is relatively low compared to other fat-soluble vitamins. Besides the liver, vitamin K can be detected in other organs such as heart and pancreas (3, 6).

**Fig. 1:**
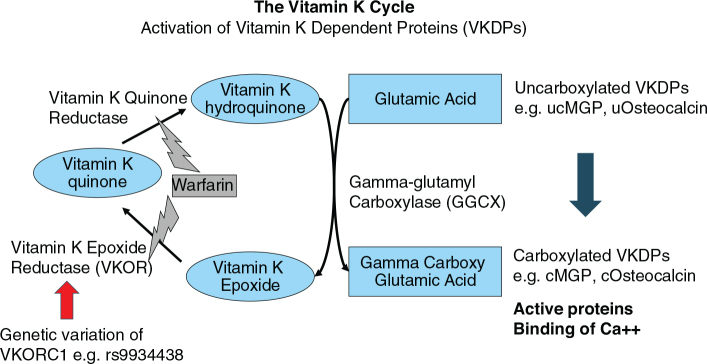
The vitamin K cycle.

The primary physiological role of vitamin K is post-translational activation of VKDPs. The best studied are the coagulation proteins that are crucial to homeostasis. Vitamin K (in the form of vitamin K hydroquinone) is a necessary co-factor for gamma-carboxylation of VKDPs by the enzyme gamma-glutamyl carboxylase (GGCX). Vitamin K hydroquinone becomes oxidized to vitamin K epoxide that needs to be reduced to vitamin K quinone by the enzyme vitamin K epoxide reductase (VKOR). VKOR is thus important to recycling of vitamin K (known as the vitamin K cycle; see Fig. 1) and has been shown to be crucial for the function of coagulation factors and homeostasis.

Bleeding due to insufficient dietary intake of vitamin K is extremely rare in otherwise healthy individuals. Vitamin K transport across the placenta is poor and vitamin K content is low in breast milk. Bleeding episodes due to vitamin K deficiency in new-born infants, especially those born prematurely, were not rare before implementation of vitamin K prophylaxis in new-borns.

Vitamin K is suggested to have a possible role in the prevention of several age-related processes such as progression of bone loss and vascular calcification (7). Vitamin K may have a role in progression of vascular calcification. The possible mechanism is activation of gamma-carboxyglutamic protein (MGP), which is dependent on vitamin K.

Generally, little is known about differences between the different vitamin K forms with regard to their physiological functions. It is not clear whether menaquinones are more effective than phylloquinone in terms of their capacity for carboxylation (activation) of VKDPs (8).

There are many potential sources of individual variation in metabolism of vitamin K. They include genetic variation in various biochemical pathways, of which the best established is a genetic variant in the VKOR encoding gene that renders carriers more sensitive to low dietary intake of vitamin K (4, 9) (Fig. 1). Individual variation in the gut microbiota could also cause variation in gut bacterial production of menaquinones, but it is not known to what extent gut bacterial production plays a role in human physiology and health. Finally, there are specific drugs that antagonize VKOR function, the best known example being a type of anticoagulants (e.g. warfarin and phenprocoumon).

## Assessment of nutrient status

There are several biomarkers that reflect vitamin K intake; however, none are considered sufficient to be used alone (10, 11).

Serum concentration of phylloquinone is a short-term marker of phylloquinone intake, and it is the most frequently used marker for vitamin K status (3, 10–12). Serum phylloquinone concentration responds to dietary changes. Samples for measurement should be collected after fasting and the results should be adjusted for serum triglycerides, as phylloquinone is transported in triglyceride-rich lipoproteins (10). Even though there are suggestions for a threshold, there is no consensus on what serum phylloquinone concentration would indicate adequate intake (10, 11).

Menaquinones are not typically detected in circulation in individuals consuming a Western diet, unless menaquinone supplements are taken (10, 11). The clinical significance of measurement of serum menaquinones remains unknown.

Indirect methods to assess vitamin K status include coagulation assays, undercarboxylated VKDPs and urinary vitamin K metabolites. Global coagulation assays (prothrombin time and partial thromboplastin time) are not sensitive markers of vitamin K intake within the range of typical dietary intakes (11). Furthermore, they are not specific to vitamin K intakes as different diseases can affect their levels.

Vitamin K is needed for carboxylation of hepatic and extrahepatic proteins. Therefore, low intake of vitamin K could increase the proportion of undercarboxylated (inactive) forms of these proteins. Undercarboxylated prothrombin (protein induced by vitamin K absence/antagonism, PIVKA-II) is measurable in circulation and responds to dietary changes in vitamin K intake. Osteocalcin (OC) is a VKDP synthesized in bone. Vitamin K depletion increases the undercarboxylated fraction of OC (ucOC) in serum. ucOC should be expressed as the proportion of total OC. Both PIVKA-II and ucOC are suggested to be used as indicators of vitamin K status (10, 11). MGP is vitamin K-dependent protein that has a function in inhibiting calcification in the vascular tissue and cartilage. There are multiple isoforms of ucMGP and it is suggested that defosforylated-ucMGP increases with vitamin K deficiency (8, 13). ucMGP should also be expressed as fraction of total MGP. There is experimental evidence from human studies that levels of the undercarboxylated VKDPs (ucOC, ucMGP, and PIVKA-II) decrease after vitamin K supplementation (3). Thus, undercarboxylated VKDPs hold potential for use as biomarkers of vitamin K status and estimation of DRVs. However, it is generally agreed that more research is needed, e.g. on dose-response, optimal level of gamma-carboxylation, relationships with health outcomes and what biomarker to choose.

There are also urinary markers of vitamin K intake and metabolism (10, 11). Gamma-carboxyglutamic acid is produced in turnover of VKDPs and it is excreted to urine. Five carbon and seven carbon aglycone metabolites are produced in vitamin K catabolism. Menadione is an intermediate product when phylloquinone is converted to menaquinone-4. Menadione is also detectable in urine. All these urinary markers are suggested to respond to dietary changes in vitamin K intake. However, data are limited and clinical significance unknown. Further limitation is that reliable measurement would need 24 h urine collection.

## Dietary intake in Nordic and Baltic countries

Phylloquinone is plant-based and its sources include leafy green vegetables, and certain vegetable oils (soybean, canola/rapeseed and olive oils) and fat spreads made from the oils (3, 14–16). Menaquinones-5 through -13 have a bacterial origin and their main sources include fermented foods, meat and dairy products. Menaquinone-4 is formed from phylloquinone or other menaquinone forms (17). Dietary sources of menaquinone-4 include meat and dairy products. Phylloquinone is generally regarded as the predominant form of vitamin K in the Western diets; however, the limited knowledge on menaquinone contents in foods complicates the dietary intake assessment (3).

There is very little up-to-date information on vitamin K intake from nationally representative samples in Nordic and Baltic countries. Furthermore, data for different age groups is very limited. Intake of total vitamin K (phylloquinone and menaquinones) is estimated to be 115 µg/d in adult men and 110 µg/d in adult women in Finland (18). The total vitamin K intake is estimated to be 286 µg/d in adult men and women in Latvia (19). Both studies evaluated dietary intake using two 24 h recalls. In Sweden, based on a market basket study, the availability of vitamin K (phylloquinone + menaquinone-4) was estimated to be 184 µg/d (20). Earlier data on vitamin K intakes in Nordic and Baltic countries has been reviewed by EFSA (3). Results on dietary intake should be interpreted cautiously as food composition databases may have limitations and vitamin K content data mostly include only phylloquinone, not menaquinones (14, 16). In addition, the relative bioavailability of different forms of vitamin K is poorly known and limits the interpretation of summed intakes of different vitamin K forms.

EFSA recommends that an adequate intake of phylloquinone is 1 µg/kg body weight per day for all age and sex population groups (3). That corresponds to 70 µg/day for all adults. Adequate intake of vitamin K is set to 90 µg/d in adult women and 120 µg/d in adult men by Food and Nutrition Board at the National Academies of Sciences, Engineering, and Medicine (NASEM, previously Institute of Medicine) in the USA (21).

## Health outcomes relevant for Nordic and Baltic countries

### Deficiencies

Bleeding and haemorrhage are the classic signs of vitamin K deficiency affecting coagulation. Vitamin K deficiency in adults is rare and usually limited to people with malabsorption disorders or those taking drugs, e.g. vitamin K antagonists, which interfere with vitamin K metabolism (11).

In infants, vitamin K deficiency can develop and factors that contribute to it include poor placental transfer, low vitamin K content in breast milk, low activity of enzymes in vitamin K cycle and poor intestinal absorption (22). The syndrome is known as vitamin K deficiency in new-borns.

### Toxicities

No toxic effects have been reported. High doses (45–90 mg/d) of menaquinone-4 have resulted in adverse effects including gastrointestinal disorders and skin lesions in some studies; however, the effects were thought not to be serious (23, 24). Menaquinone-7 has been evaluated to be safe when ingested as a dietary supplement with doses ranging from approximately 50 µg/d up to 600 µg/d (15). Revision of tolerable upper intake level (UL) for vitamin K was not part of the opinion by EFSA panel (3), and previously Scientific Committee on Food concluded that the evidence is too limited to set an UL (25).

### Cardiovascular diseases

Vitamin K’s potential preventive effect against vascular calcification is believed to at least partly be mediated via vitamin K dependent activation of MGP. Meta-analyses of observational studies found that low vitamin K status is associated with higher risk of cardiovascular disease and mortality (26). Although observational studies may be able to provide evidence of long-term effects on incidence of CVD, they may be prone to biases, e.g. confounding and inaccurate assessment of vitamin K status/intake.

A limited number of randomized controlled trials (RCTs) of the effect of vitamin K supplementation on CVD risk have been performed, primarily in high-risk populations, e.g. chronic kidney disease patients, using intermediate CVD endpoints such as arterial stiffness and vascular calcification. To date, results have been conflicting, which may be due to variation in form and dose of vitamin K, length of intervention period, and outcome measures (27). Some benefit has been indicated in subgroups with existing calcification at baseline.

At this point, the preventive effect of vitamin K on CVD is uncertain, and there is a need for more RCTs with sufficient power, duration and follow-up in both low and high-risk populations.

### Type 2 diabetes

Although observational studies have found that low vitamin K status tends to associate with biomarkers of impaired glucose regulation, caution should be exercised as many co-morbidities and lifestyles could confound these associations. Systematic reviews of RCTs in humans assessing the effects of vitamin K supplementation on various glucose and insulin sensitivity indices have not consistently confirmed a beneficial effect of vitamin K (28, 29). More RCTs are needed to investigate the possible effects of vitamin K on glucose homeostasis in both healthy and diabetic individuals before conclusions can be drawn.

### Cancers

Vitamin K is suggested to have anticancer effects; however, evidence is limited to cell studies (30).

### Osteoporosis

A meta-analysis of observational studies suggested that higher dietary phylloquinone intake could modestly reduce the risk of fractures (31). A meta-analysis of RCTs reported the effect of phylloquinone and menaquinone supplements (doses ranged from 100 µg to 45 mg daily) on bone mineral density and fracture risk (32). Vitamin K supplementation appeared to have little effect on bone mineral density in postmenopausal or osteoporotic patients. A small reduction in the risk of clinical fractures was reported with no effect on vertebral fracture. More research is needed as many studies are conducted in postmenopausal women limiting the generalizability of the results, and many trials are rated as having high risk of bias. Of note is that some initial Japanese studies showing protective effects have been retracted (33, 34).

### Other health outcomes

Vitamin K is suggested to have a role in brain function (35) and lung diseases (36). However, there is very limited evidence to support effects of vitamin K on these health outcomes.

## Requirement and recommended intakes

### Main findings

Vitamin K is known to function as an enzymatic cofactor in the gamma-carboxylation of VKDPs. However, the amount of vitamin K needed for optimal functioning of the different VKDPs is not known. There are several limitations in the knowledge regarding vitamin K which hinder the setting of DRV for vitamin K.

### Recommendations

For prevention of vitamin K deficiency bleeding, all new-born infants should receive vitamin K prophylaxis; intramuscular administration is suggested to be recommendable even though evidence is of low quality (37).

In NNR 2012, a provisional recommended intake of 1 µg phylloquinone/kg body weight per day was given for both children and adults. Similar recommendation on adequate intake of phylloquinone has been set by the EFSA (3).

There is limited data available on the need of vitamin K during pregnancy and lactation and health outcomes during pregnancy (3, 38).

### Limitations of the scoping review

There were no qualified SRs available for vitamin K. In addition, the number of SRs and meta-analysis is limited, and they cover mostly cardiovascular and bone-related outcomes. Evidence on different health outcomes still relies a lot on observational studies and more high quality RCTs are needed. Studies on vitamin K include mostly adult or older participants.

### Data gaps for future research

More knowledge is needed in order to give more evidence-based recommendations for vitamin K intake. More research is needed on the absorption, metabolism, storage and functions of the various forms of vitamin K (and their differences) including the possible role of menaquinones produced by the gut microbiota. Development and validation of biomarkers of vitamin K status and functions are important both for improving our knowledge of functions of vitamin K and potentially deriving cut-offs for requirements.

Food composition databases generally do not contain precise data on vitamin K contents of all foods, particularly for menaquinones data are lacking. More complete food composition data are required for better assessment of vitamin K intakes in populations and the long-term health effects of vitamin K intake in observational epidemiological studies. It is important to note that many suggested associations between vitamin K and disease outcomes are based on observational studies and ultimately intervention studies in humans are needed to confirm claimed novel health effects of vitamin K beyond coagulation. Unfortunately, this is a long-term effort involving large study populations assessing incidence of disease or their accepted intermediate objective markers. Finally, studies on genetic variation of vitamin K metabolism and effects could contribute to our understanding of the functions and variation in requirements of vitamin K.
